# Mitochondrial Dysfunction in Parkinson’s Disease: New Mechanistic Insights and Therapeutic Perspectives

**DOI:** 10.1007/s11910-018-0829-3

**Published:** 2018-04-03

**Authors:** Jin-Sung Park, Ryan L. Davis, Carolyn M. Sue

**Affiliations:** 1 Department of Neurogenetics, Kolling Institute, University of Sydney and Northern Sydney Local Health District, St. Leonards, Sydney, NSW 2065 Australia; 2 Sydney Medical School-Northern, University of Sydney, St. Leonards, Sydney, NSW 2065 Australia; 3 Department of Neurology, Royal North Shore Hospital, Northern Sydney Local Health District, St. Leonards, Sydney, NSW 2065 Australia

**Keywords:** Parkinson’s disease, Neurodegeneration, Mitochondria, Bioenergetics, Mitophagy, Mitochondrial biogenesis, Therapy

## Abstract

**Purpose of Review:**

Parkinson’s disease (PD) is a complex neurodegenerative disorder, the aetiology of which is still largely unknown. Overwhelming evidence indicates that mitochondrial dysfunction is a central factor in PD pathophysiology. Here we review recent developments around mitochondrial dysfunction in familial and sporadic PD, with a brief overview of emerging therapies targeting mitochondrial dysfunction.

**Recent Findings:**

Increasing evidence supports the critical role for mitochondrial dysfunction in the development of sporadic PD, while the involvement of familial PD-related genes in the regulation of mitochondrial biology has been expanded by the discovery of new mitochondria-associated disease loci and the identification of their novel functions.

**Summary:**

Recent research has expanded knowledge on the mechanistic details underlying mitochondrial dysfunction in PD, with the discovery of new therapeutic targets providing invaluable insights into the essential role of mitochondria in PD pathogenesis and unique opportunities for drug development.

## Introduction

The key manifestations associated with a clinical diagnosis of Parkinson’s disease (PD) are motor deficits resultant of focal dopaminergic nigral neurodegeneration. However, these manifestations appear late in the disease course, with mounting evidence indicating seminal pathogenic events occur a decade or more prior [1]. Prevailing theory holds that PD progression is largely mediated by pathological protein aggregation that is either the cause or corollary of dysfunction in multiple interrelated cellular pathways [2].

PD is now widely accepted as a complex, multifactorial disease that can have diverse genetic, biological and environmental influences [3]. Although sporadic PD patients, who lack evidential family history and a definitive genetic basis, account for > 90% of disease cases, the familial forms of PD have inferred cellular pathways central to PD pathophysiology [4]. With the majority of genetic PD loci directly associated with mitochondria, mitochondrial dysfunction has been implicated as an integral disease component [5]. This review focuses on recent advances in understanding of the role that mitochondrial dysfunction plays in the pathogenesis of both sporadic and familial PD.

## Mitochondrial Dysfunction in Familial Parkinson’s Disease

**Fig. 1:**
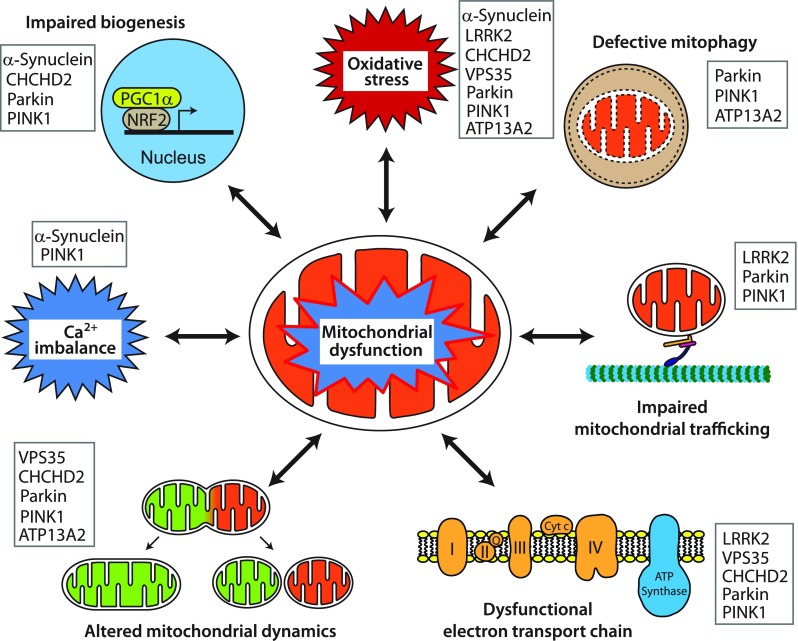
Representative pathways of mitochondrial dysfunction involved in Parkinson’s disease pathophysiology. Mitochondrial dysfunction associated with PD pathogenesis can result from impairment of mitochondrial biogenesis, increased reactive oxygen species production, defective mitophagy, compromised trafficking, electron transport chain dysfunction, variations to mitochondrial dynamics, calcium imbalance or combinations thereof. The potential complex interplay of the various functions leads to a vicious cycle of progressive cellular dysfunction that ultimately results in neurodegeneration that underlies PD pathogenesis and progression. Proteins mentioned in this review that contribute pathologically to the different pathways are listed

To date, a handful of genes have been identified as monogenic causes of familial PD, with many of the pathogenic mutations in these genes directly linked to mitochondrial dysfunction (i.e. autosomal dominant *SNCA* and *LRRK2* mutations and autosomal recessive *Parkin*, *PINK1* and *ATP13A2* mutations) [6]. More recently, new roles in the regulation of mitochondrial biology have been determined for these genes, and new PD genes associated with mitochondrial (dys)function, such as *VPS35* and *CHCHD2*, have been identified, further underpinning the essential role of mitochondrial function to the aetiology of PD (Fig. 1).

### Autosomal Dominant PD

#### *SNCA*

α-Synuclein (α-Syn) is a small 140 amino acid polypeptide, encoded by *SNCA*. Although its function is still largely unknown, it has been reported to mediate neurotransmitter release at presynaptic terminals and interact with membranes of various organelles, including mitochondria. Indeed, α-Syn has a non-canonical mitochondrial targeting sequence, and has been localised to mitochondrial membranes and shown to influence mitochondrial structure and function [7].

α-Syn was initially linked to PD as the main component of Lewy bodies, with *SNCA* later identified as the first genetic familial PD gene [8•]. Increased levels of wild-type (WT) α-Syn and, to a greater extent, α-Syn with PD-linked mutations, such as A53T, E46K and H50Q, induce mitochondrial fragmentation and reactive oxygen species (ROS) production in vitro and in vivo [9]. Furthermore, α-Syn was recently localised to mitochondria-associated membranes (MAM), a specialised structure forming an interface between the endoplasmic reticulum (ER) and mitochondria that is important for regulating Ca^2+^ signalling and apoptosis. Pathogenic mutations in α-Syn were found to reduce binding to MAM and increased mitochondrial fragmentation, suggesting a role for α-Syn in regulating mitochondrial morphology [10]. For example, overexpressed WT or mutant α-Syn was found to cause dissociation of ER and mitochondria at MAM, thereby impairing Ca^2+^ exchange and reducing mitochondrial energy production [11].

In addition to direct effects on mitochondrial morphology, a recent study showed that α-Syn can influence mitochondrial biogenesis via regulation of peroxisome proliferator-activated receptor gamma coactivator 1-α (PGC1α). In this study, treatment of human DA neurons carrying A53T with mitochondrial toxins induced S-nitrosylation of the transcription factor myocyte-specific enhancer factor 2C (MEF2C), leading to a reduction in mitochondrial biogenesis via downregulation of PGC1α [12].

#### *LRRK2*

Mutations in *Leucine Rich Repeat Kinase 2* (*LRRK2*) cause a variably penetrant autosomal dominant form of PD and have been identified as the most common cause of familial PD [6]. LRRK2 is a multifunctional protein kinase and LRRK2 mutants are known to exert their pathogenic action via increased kinase activity. Various models overexpressing WT or PD-associated mutant LRRK2 have shown increased vulnerability to mitochondrial toxins, along with defects in mitochondrial dynamics and increased ROS production (reviewed in [9]). Consistently, physiological levels of the common LRRK2 G2019S mutant were found in association with mitochondrial abnormalities in patient-derived dopaminergic neurons [13], as well as knock-in mice [14].

**Fig. 2:**
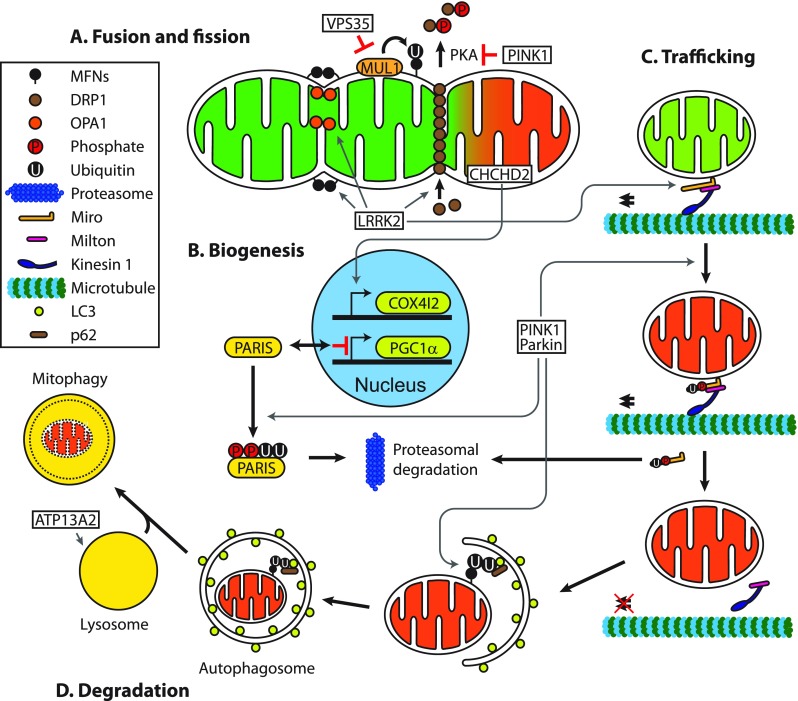
Mitochondrial function of Parkinson’s disease-related proteins. A. VPS35 mediates degradation of mitochondrial E3 ubiquitin ligase 1 (MUL1), which ubiquitinates mitofusins (MFNs), acting as a pro-fusion factor. Conversely, PINK1 inhibits protein kinase A (PKA) mediated release of dynamin-related protein 1 (DRP1) from mitochondria, promoting mitochondrial fission. Additionally, LRRK2 acts on several fission and fusion effectors, such as MFNs, optic atrophy 1 (OPA1) and DRP1, to variably alter the balance of mitochondrial dynamics. B. Parkin interacting substrate (PARIS) inhibits mitochondrial biogenesis by suppressing expression of the master regulator peroxisome proliferator-activated receptor gamma coactivator 1-α (PGC1α). Under steady-state conditions, PINK1 and Parkin mediate the degradation of PARIS by phosphorylation and ubiquitination respectively, followed by proteasomal degradation, which maintains PGC1α levels and mitochondrial biogenesis. Under mitochondrial stress, CHCHD2 translocates to the nucleus and upregulates expression of mitochondrial complex IV subunit 4 isoform (COX4I2). C. Miro facilitates mitochondrial transportation with another adaptor protein Milton and the motor protein Kinesin-1. PINK1 and Parkin promote mitophagy of dysfunctional mitochondria by inducing proteasomal degradation of Miro and thereby halting mitochondrial transport. Similarly, LRRK2 has been shown to facilitate removal of Miro. D. Parkin, activated by PINK1, ubiquitinates outer mitochondrial membrane proteins, such as MFNs, to which the autophagosomal protein microtubule-associated protein light chain 3 (LC3) binds with p62, a polyubiquitin-binding protein, leading to engulfment of dysfunctional mitochondria by autophagosomes. Degradation of mitochondria occurs upon fusion with lysosomes. ATP13A2 ensures mitophagy by maintaining functional lysosomes

Several proteins are known to interact with LRRK2 and mediate pathological effects on mitochondria. For instance, the mitochondrial fission protein, dynamin-related protein 1 (DRP1), has been shown to function as an effector of mitochondrial fragmentation through LRRK2-mediated phosphorylation at S616 [15] (Fig. 2). Moreover, LRRK2 appears to interact with other fission/fusion proteins, such as mitofusin (MFN) 1/2 and optic atrophy 1 (OPA1) [16]. LRRK2-mediated increased proton leak and loss of mitochondrial membrane potential (ΔΨm) are likely caused by upregulation of mitochondrial uncoupling protein (UCP) 2 and UCP4 [17]. In addition, mutant LRRK2 contributes to defective mitophagy by interfering with mitochondrial trafficking, as G2019S has been shown to impair proteasomal degradation of Miro, an outer mitochondrial membrane (OMM) protein that tethers mitochondria to microtubule motor proteins, and thereby mitophagy by disrupting the interaction between LRRK2 and Miro [18].

#### *VPS35*

The association between vacuolar protein sorting-associated protein 35 (VPS35) and PD was first observed in European PD cohorts with a family history suggestive of an autosomal dominant inheritance [19, 20]. VPS35 is a core component of the retromer complex that mediates retrograde delivery of cargo from endosomes to Golgi, as well as recycling cargo from endosomes to the cell surface [21]. Early studies reported that PD-associated mutations in *VPS35* conferred vulnerability to the mitochondrial toxin 1-methyl-4-phenylpyridinium (MPP^+^) in vitro [22].

The main function of VPS35 in mitochondria seems to be in regulating mitochondrial dynamics through interaction with mitochondrial fission/fusion proteins. Recent studies have shown that mutant VPS35 can trigger mitochondrial fragmentation, which leads to neurodegeneration. This occurs through either a decrease in the degradation of mitochondrial E3 ubiquitin ligase 1 (MUL1), which in turn increases MFN degradation [23] (Fig. 2), or by enhancing the turnover of DRP1 complexes via mitochondrial-derived vesicle-dependent trafficking to lysosomes [24]. Also, increased mitochondrial fragmentation caused by the VPS35 mutation D620N was shown to impair mitochondrial complex I assembly and activity [25].

#### *CHCHD2*

Recently, mutations in *coiled-coil-helix-coiled-coil-helix domain containing 2* (*CHCHD2*) have been identified as a cause of autosomal dominant, late-onset PD in three Japanese families [26•]. CHCHD2 is a mitochondrial intermembrane space protein with a dual function in the mitochondria and nucleus. Under normal conditions, CHCHD2 mainly exists in mitochondria bound to mitochondrial complex IV and reduced expression of CHCHD2 has consistently been shown to decrease mitochondrial complex IV activity, with resulting increases in ROS production and mitochondrial fragmentation [27]. Intriguingly, CHCHD2 was found to translocate into the nucleus and function as a transcription factor under stress conditions, regulating the expression of mitochondrial complex IV subunit 4 isoform (*COX4I2*) [27] (Fig. 2). Furthermore, *Drosophila* deficient of CHCHD2 [28] or expressing PD-associated mutants [29] also displayed structural and biochemical mitochondrial abnormalities leading to dopaminergic neurodegeneration and motor dysfunction. These findings strongly suggest that mutations in CHCHD2 lead to nigrostriatal neurodegeneration and PD by impairing mitochondrial function.

### Autosomal Recessive PD

#### *Parkin*

Mutations in *Parkin* are the most frequent cause of autosomal recessive PD [30•], with over 120 pathogenic mutations identified so far [6]. Parkin is a cytosolic E3 ubiquitin ligase that ubiquitinates target proteins for signalling or proteasomal degradation. Parkin primarily functions in association with mitochondria, as Parkin-deficient models show profound defects in mitochondrial morphology and function [31]. Consistently, ubiquitylome analysis has revealed that the majority of Parkin targets are localised to mitochondria [32].

Parkin has diverse functions in maintaining healthy mitochondria by regulating their biogenesis and degradation via mitophagy (reviewed in [33]). In the early stages of mitochondrial degradation, Parkin is recruited to damaged or dysfunctional mitochondria and activated by PTEN-induced putative kinase 1 (PINK1), another PD-related protein (see below), leading to ubiquitination of OMM proteins and subsequent proteasomal degradation (Fig. 2). The process of mitophagy removes dysfunctional mitochondria from the healthy mitochondrial pool and facilitates their degradation via the autophagy-lysosomal pathway. Despite recent literature broadening the detailed mechanism by which Parkin mediates mitophagy in vitro, relevance to disease pathogenesis has been controversial given the lack of evidence that Parkin mediates mitophagy in vivo. However, recent studies have demonstrated endogenous Parkin-mediated mitophagy in the distal axons of rodent neurons [34] and in age-related dopaminergic neurodegeneration accompanying PD-linked motor symptoms in Parkin knockout mice with defective mitochondrial DNA replication [35•]. These findings further highlight the pathophysiological significance of Parkin-mediated mitophagy in PD over the insights obtained from in vitro models. In addition, newly developed transgenic mouse models expressing mitophagy reporters such as mt-Keima [36•] and mito-QC [37•] have finally made it possible to monitor mitophagy in mammalian brain, promising to unravel the long-standing mystery surrounding mitophagy in vivo.

Besides function in mitophagy, Parkin is known to maintain the functional mitochondrial pool by regulating mitochondrial biogenesis [31]. Under homeostatic conditions, Parkin mediates the degradation of parkin interacting substrate (PARIS), a repressor of PGC1α activity, leading to nuclear translocation of PGC1α and transcriptional activation of mitochondria-associated genes [38] (Fig. 2). Consequently, loss of Parkin function allows PARIS to accumulate and repress mitochondrial biogenesis, resulting in reduced mitochondrial mass and functional defects [39]. These findings highlight the pivotal role Parkin plays in modulating the balance of mitochondrial production and destruction.

#### *PINK1*

Mutations in *PINK1* are the second most common cause of autosomal recessive early-onset PD [6, 40•]. PINK1 is a mitochondrial serine/threonine kinase that plays a crucial role in maintaining mitochondrial homeostasis. Loss of PINK1 impairs various aspects of mitochondrial biology, including degradation, morphology and trafficking. The most widely studied of these is the function of PINK1 in mitophagy; facilitating removal of damaged mitochondria by recruiting and activating Parkin [33, 41]. PINK1 activates Parkin by a twofold mechanism: (1) direct phosphorylation of Parkin at S65 [42] and (2) trans-activation by phosphorylation of ubiquitin at S65 and subsequent binding to Parkin [43•, 44•, 45•]. In addition, PINK1 can mediate mitophagy in a Parkin-independent manner by recruiting nuclear dot protein 52 kDa (NDP52) and optineurin (OPTN) [46]. Furthermore, in a similar manner to LRRK2, PINK1 has been shown to promote mitophagy by terminating mitochondrial trafficking through phosphorylation and Parkin-mediated proteasomal degradation of Miro [47] (Fig. 2).

Loss of PINK1 has been shown to induce a wide range of mitochondrial dysfunction in cell models, *Drosophila* and mice. This is largely a result of the loss of PINK1/Parkin-mediated mitophagy, but PINK1 also regulates mitochondrial homeostasis in a number of other ways [31]. For instance, PINK1 deficiency has been found to result in mitochondrial Ca^2+^ overload [48], and the specific reduction of mitochondrial complexes I and III [49]. On the other hand, PINK1 has been shown to enhance mitochondrial fission by increasing protein kinase A (PKA)-mediated DRP1 activation [50] and to modulate mitochondrial biogenesis via regulating Parkin-mediated degradation of PARIS [51] (Fig. 2).

#### *ATP13A2*

Mutations in *ATP13A2* cause Kufor-Rakeb syndrome (KRS), a rare form of autosomal recessive juvenile-onset PD [52]. *ATP13A2* encodes a type P5B ATPase, which mainly localises to the endo/lysosomal compartment. Although ATP13A2 is believed to transport cations across organellar membranes, its transporting activity is yet to be fully defined. Nonetheless, loss of ATP13A2 in patient-derived cells shows increased susceptibility to several cations including Zn^2+^ and Mn^2+^, indicating a role for ATP13A2 in regulating these metals [52]. The association of ATP13A2 with mitochondrial function was first implicated by observation of mitochondrial dysfunction in KRS patient-derived skin fibroblasts [53]. Consistently, several studies employing ATP13A2-deficient cell models have comprehensively shown underlying mitochondrial dysfunction, including reduced ATP production, increased mitochondrial fragmentation and increased ROS production [54, 55]. In addition, loss of ATP13A2 was also found to impair glycolysis, which aggravated mitochondrial dysfunction, suggesting a broader impact of ATP13A2 deficiency on cellular bioenergetics [56].

Existence of ATP13A2 outside mitochondria led to speculation that ATP13A2 may indirectly regulate mitochondrial function. Indeed, loss of ATP13A2 has been shown to cause Zn^2+^ dyshomeostasis by impairing vesicular sequestration, leading to mitochondrial dysfunction [55]. Also, dysregulated Zn^2+^ metabolism causes lysosomal dysfunction [57], which may contribute to defective mitophagy (Fig. 2), highlighting the complex interplay between closely associated cellular pathways known to be involved in the pathogenesis of PD.

## Mitochondrial Dysfunction in Sporadic Parkinson’s Disease

Sporadic PD occurs as a seemingly random occurrence due to undetermined genetic or environmental bases in the absence of an obvious family history. It is well established that PD is a multifactorial disorder caused by impaired cellular functions that impact upon interrelated pathways and create complex feedback cycles leading to neurodegeneration [2]. Broadly, affected cellular pathways include proteostasis, oxidative stress and the multiple pathways relating to mitochondrial function (Fig. 1) [58], all of which are evident in sporadic PD.

### Genetic and Environmental Influences on Sporadic PD

It is increasingly apparent that environmental and genetic aspects contribute to PD, with combinatorial insults being more pathological than either individually [3]. Phenotypes consistent with sporadic PD can be induced by a number of endogenous and exogenous inhibitors of mitochondrial function, including rotenone, 1-methyl-4-phenyl-1,2,3,6-tetrahydropyridine (MPTP), paraquat, nitric oxide, the dopamine metabolite aminochrome and others [3, 9]. Consistently, an increased risk for developing idiopathic PD has been demonstrated in rural populations exposed to agricultural pesticides and herbicides [59] and a significantly younger onset of sporadic PD has been linked to chronic occupational exposure to pesticides and heavy metals [60]. From a genetic perspective, Genome-Wide Association Studies (GWAS) have provided evidence that polymorphisms in familial PD genes are risk factors for developing sporadic PD, linking the pathogenesis of familial and sporadic PD [61]. Sporadic PD risk has been attributed to a number of loci including regions of as yet unknown influence [62•, 63], as well as familial PD genes associated with mitochondrial dysfunction, e.g. *Parkin*, *PINK1*, *ATP13A2*, *CHCHD2*, *SNCA*, *LRRK2* and *GBA* [64].

### α-Synuclein in Sporadic PD

α-Syn has been found to bind to OMM proteins, such as voltage-dependent anion-selective channel 1 (VDAC1), translocase of outer membrane (TOM) 40 and TOM20, and mediate mitochondrial dysfunction [65]. Furthermore, VDAC1 levels were found to be reduced in sporadic PD patient nigral neurons in association with α-Syn aggregations and is therefore implicated as a component of overall mitochondrial dysfunction in sporadic PD [66]. This may be mediated via the α-Syn-induced activation of the mitochondrial permeability transition pore, which depolarises the mitochondrial membrane potential leading to mitochondrial fragmentation and degradation. In the setting of dysfunctional mitophagy and trafficking (as discussed above), this would be expected to enhance cellular dysfunction and death [66]. Furthermore, α-Syn pathogenicity is related to aggregation rather than a loss of intrinsic function, with the formation of Lewy bodies, chiefly comprised of α-Syn, a hallmark of neuronal degeneration in sporadic PD [67]. Aggregated α-Syn affects proteostasis by impairing the function and trafficking between ER, Golgi and the autophagy-lysosomal system, as well as impacting on mitochondrial functions including energy production, calcium and iron buffering and ROS production.

### Iron Accumulation and Oxidative Stress

Oxidative stress is intimately linked to mitochondrial (dys)function, with mitochondria producing ~ 90% of cellular ROS [68]. It is apparent that synucleinopathy, oxidative stress and mitochondrial dysfunction are locked in a vicious interdependent feedback cycle in sporadic PD [58], with mitochondrial accumulation of α-Syn inhibiting complex I activity and driving ROS production via the consequent respiratory chain dysfunction [7]. In particular, iron accumulation observed in the substantia nigra of sporadic PD patient brain causes increased ROS production, transcriptional upregulation of *SNCA* and increased α-Syn aggregation [69, 70]. Mitochondria have active iron exchange with the cytoplasm, required for the synthesis of iron sulphur clusters, which are integral components of complex I and II and sensitive to oxidative stress. Consequently, inhibition of complex I by rotenone, MPTP and paraquat poisoning have been shown to result in iron accumulation in association with PD [71]. Inhibition of the ubiquitin proteasome system also causes cellular iron dyshomeostasis, further adding to the positive feedback on ROS generation and α-Syn aggregation [72]. In addition, neuronal iron accumulation impacts on mitochondrial reticular connectivity, as shown for calcineurin-dependent effects on DRP1 [73] and calcium release via Ryanodine receptors [74].

### Mitochondrial Quality Control

The mitochondrial quality control mechanisms of dynamic complementation in concert with balanced mitophagy and biogenesis work to maintain a healthy cellular mitochondrial pool and bioenergetic function under steady-state conditions [33], reasoning why disruption of these pathways cause mitochondrial dysfunction that underlies PD pathogenesis.

An emerging area of interest is the influence that lipids and lipid pathways have on PD. For instance, the master regulator of lipogenesis, sterol regulatory element binding transcription factor 1 (*SREBF1*), was identified by GWAS as a risk locus for sporadic PD [75] and was subsequently validated by genome-wide RNAi screening as a regulator of Parkin-mediated mitophagy [76]. This was further endorsed by administration of genistein, an inhibitor of sterol regulatory element binding protein (SREBP) activation, which blocked Parkin recruitment to mitochondria and was partially rescued by exogenous lipid supplementation, thereby providing a mechanistic link between lipid synthesis and mitophagy and filling an evidence gap for the association of mitophagy with sporadic PD [77].

The importance of mitophagy in PD pathogenesis is evident from the prevalence of familial cases associated with *PINK1* and *Parkin* mutations. However, cytoplasmic hybrid cells generated from sporadic PD patient platelets were also found to have fragmented mitochondrial networks [15]. This was the result of fusion impairment due to proteolysis of OPA1 and fission enhancement by phosphorylation of DRP1 S616. In a more recent study using neurotoxin models of sporadic PD, it was shown that increased nitric oxide levels caused the nitrosylation of Parkin, impairing its ubiquitin ligase activity and resulting in an upregulation of phosphorylated S616 DRP1 recruitment to mitochondria and consequential mitochondrial hyper-fragmentation [78]. This study contextualised the role of DRP1 in mitochondrial fragmentation and dysfunction that leads to neuronal cell death in sporadic PD and identified nitrosylated Parkin as a possible therapeutic angle.

Cargo trafficking along axonal microtubules is important for shuttling cellular components to and from the synaptic terminals. Of particular interest for the pathogenesis of sporadic PD is the axonal trafficking of α-Syn and mitochondria [79]. Dysfunctional trafficking has been linked to sporadic PD by a number of mechanisms, including a reduction in motor protein expression with consequent accumulation of α-Syn in the axons and soma [80•], as well as decreased degradation of the mitochondrial-molecular motor tether Miro and consequent impairment of mitochondrial motility (also a feature in familial PD; see above) [18]. Additionally, LRRK2 and Parkin recruitment to mitochondria was impaired upon CCCP treatment in sporadic PD patient-derived fibroblasts, highlighting that the LRRK2/DRP1 and PINK1/Parkin pathways act in parallel, converge on Miro and are impaired in sporadic PD [18].

It has been identified that expression of PGC1*α* is reduced in sporadic PD brain [81•] and can be reduced by direct binding of accumulated α-Syn to the *PPARGC1A* promoter in the setting of oxidative stress [82] or by methylation of the *PPARGC1A* promoter [83]. On the converse, PGC1*α* expression has been shown to mitigate α-Syn oligomerisation [81•] and protect DA neurons [84]. These findings indicate that PGC1*α*-mediated mitochondrial biogenesis imparts neuroprotection that becomes compromised in the setting of sporadic PD.

### Dysfunctional Electron Transport Chain and Alterations to the Mitochondrial Genome

Since the original observation of MPTP causing mitochondrial dysfunction in PD, mitochondrial complex I has been considered central to the pathogenesis of PD. However, one question that arises when considering complex I in PD is, why do mitochondrial disease patients with complex I deficiency rarely develop PD. To date, no mitochondrial DNA (mtDNA) mutations have been found to cause PD, despite genes integral to complex I being encoded by mtDNA. Instead, Parkinsonism associated with mitochondrial diseases is largely restricted to mutations affecting the mtDNA maintenance genes *POLG* and *TWINKLE* (encoding the mtDNA polymerase and helicase, respectively), but is inconsistently observed [85, 86]. Some insight was provided by the exonuclease dysfunctional *POLG* mutator mouse, which alone did not recapitulate a PD phenotype due to compensatory mitochondrial biogenesis, but when crossed with a Parkin knockout mouse convincingly displayed a PD phenotype [35]. This suggests accumulation of somatic mtDNA mutations is insufficient to cause PD and other insults are required to elicit disease. Nevertheless, supporting the notion of increased mtDNA mutation in PD, rotenone treatment of rats was found to increase the rate of somatic mtDNA mutation, particularly in the substantia nigra [87].

Respiratory chain enzymology in single neurons from idiopathic PD patients showed complex I and II were typically affected [88]. In addition, mtDNA from these cells showed multiple deletions on the background of a common deletion. Consistently, neuronal mtDNA copy number was found to increase with age in controls, but not in PD patients [88]. In fact, the accumulation of deleted mtDNA in PD patients meant there was wild-type mtDNA depletion, which effectively raised the relative levels of somatic mutations, likely contributing to an underlying mitochondrial bioenergetic defect in sporadic PD neurons [89]. Supporting this, sporadic PD patients show an accumulation of mtDNA mutations in the setting of reduced mtDNA copy number, predominantly in the substantia nigra [90, 91]. On this basis, as age is the greatest risk factor for developing PD and ageing is associated with a decline in mitochondrial function (which results from accumulation of mtDNA mutations, reduction in respiratory chain activity and an increase in oxidative stress that ultimately causes reduced cellular bioenergetics and favours α-Syn aggregation), it appears that mtDNA and respiratory chain based mitochondrial dysfunction contributes to PD pathogenesis by lowering the threshold for susceptibility to other genetic and environmental insults.

## Emerging Therapeutic Strategies

The common involvement of mitochondrial dysfunction in PD represents an attractive target for drug development. Accordingly, various strategies have been devised to improve mitochondrial function in both familial and sporadic PD. Enhancing mitophagy presents as an effective approach due to growing evidence for its general impairment in PD. Increasing Parkin activity by inhibiting c-Abl-mediated phosphorylation using nilotinib has been shown to be neuroprotective [92], while the ATP analog kinetin triphosphate increased mutant PINK1 activity, leading to enhanced Parkin recruitment [93]. Inhibition of deubiquitinating enzymes also increases Parkin-mediated mitophagy as ubiquitin specific peptidase (USP) 8, 15 and 30 antagonize the action of Parkin, whereas inhibition of these USPs increased mitochondrial degradation [94]. Additionally, activation of non-canonical mitophagy may provide an alternative avenue to restore mitochondrial function in PD as several proteins such as Fun14 domain-containing protein 1 (FUNDC1) and autophagy and beclin 1 regulator 1 (Ambra1) displayed an ability to modulate mitophagy in a PINK1/Parkin-independent manner [95]. In particular, Nip3-like protein X-mediated mitophagy [96] was recently found to restore mitochondrial function and prevent neurodegeneration in the setting of Parkin or PINK1 deficiency, highlighting this pathway as a potential target for therapeutic intervention.

Increasing mitochondrial biogenesis is another strategy to replenish neurons with healthy mitochondria. Dimethyl fumarate or BG-12 has been effective in phase III trials of relapsing multiple sclerosis [97] and approved for treating patients, highlighting a potential application in PD. A recent study showed that BG-12 exerts beneficial effect by increasing mitochondrial biogenesis in mice and humans via the transcription factor nuclear factor (erythroid-derived 2)-like 2 (NRF2) [98]. Another activator of the NRF2 pathway, synthetic triterpenoids, showed a protection of dopaminergic neurons against MPTP [99]. Likewise, PGC1α has been a popular target due to its potent role in inducing mitochondrial biogenesis. Bezafibrate [100] and quercetin [101] showed beneficial effects by increasing mitochondria in rodent models for neurodegeneration, proposing an opportunity for new drug development.

Mitochondrial-targeted antioxidants and flavonoids have shown promising results in animal models, and attempts to mitigate mitochondrial dysfunction using antioxidants have produced positive outcomes in preclinical settings [102]. However, recent clinical trials for creatine and coenzyme Q10 have not demonstrated disease-modifying benefit in patients with PD [103, 104], indicating that more targeted antioxidant approaches may be required or that oxidative stress is a downstream effect of mitochondrial dysfunction rather than a direct cause of PD-related neurodegeneration.

## Summary

PD is a multifactorial disease caused by combinations of genetic and environmental factors in which the balance may vary from individual to individual. Among these factors, mitochondrial dysfunction plays an integral role in the pathogenesis of PD, with accumulated evidence supporting centrality in both sporadic and familial PD. Furthermore, the discovery of new mitochondria-associated genes as causes of PD continues to expand our understanding of the molecular mechanisms underlying mitochondrial dysfunction and consequential impact on neurodegeneration. Rapid advances in such knowledge have created an unprecedented opportunity for the development of effective PD therapies by targeting mitochondrial dysfunction. Although several drug candidates have failed in recent clinical trials, cohorts have not been stratified according to these risk factors potentially offering an explanation for their lack of success. Preclinical results of other drugs targeting newly identified molecules are promising, leaving hope for future effective PD therapies. Much work remains to define the mechanisms underlying mitochondrial dysfunction and its pathogenic influence in the development of both sporadic and familial PD.
